# A Novel Method for Polar Form of Any Degree of Multivariate Polynomials with Applications in IoT

**DOI:** 10.3390/s19040903

**Published:** 2019-02-21

**Authors:** Sedat Akleylek, Meryem Soysaldı, Djallel Eddine Boubiche, Homero Toral-Cruz

**Affiliations:** 1 Department of Computer Engineering, Ondokuz Mayıs University, Samsun 55139, Turkey; sedat.akleylek@bil.omu.edu.tr (S.A.); meryem.soysaldi@bil.omu.edu.tr (M.S.); 2 LaSTIC Laboratory, Department of Sciences & Technologies, University of Batna 2, Batna 05000, Algeria; dj.boubiche@gmail.com; 3 Department of Sciences and Engineering, University of Quintana Roo, Chetumal 77019, Mexico

**Keywords:** multivariate polynomials, post-quantum cryptography, bilinear functions, identification schemes, IoT, RFID

## Abstract

Identification schemes based on multivariate polynomials have been receiving attraction in different areas due to the quantum secure property. Identification is one of the most important elements for the IoT to achieve communication between objects, gather and share information with each other. Thus, identification schemes which are post-quantum secure are significant for Internet-of-Things (IoT) devices. Various polar forms of multivariate quadratic and cubic polynomial systems have been proposed for these identification schemes. There is a need to define polar form for multivariate *d*th degree polynomials, where d≥4. In this paper, we propose a solution to this need by defining constructions for multivariate polynomials of degree d≥4. We give a generic framework to construct the identification scheme for IoT and RFID applications. In addition, we compare identification schemes and curve-based cryptoGPS which is currently used in RFID applications.

## 1. Introduction

Identification schemes are needed to provide identity of the communicating parties [1]. In these schemes, there are two parties: prover and verifier. The prover wants to prove to the verifier that he/she is really the person who is communicating. The verifier asks questions to convince the prover’s commitment. The prover tries to convince the verifier without revealing his secret information. Identification schemes are the most important elements for many area, such as devices connected to the Internet. IoT is a popular technology that allows objects to identify and communicate with each other by connecting to Internet [2]. IoT consists of billions of devices that can communicate, gather and share information, make a decision without human interaction. IoT provides the opportunities for devices to see, hear, think and talk with each other via Internet [3]. According to Cisco predictions, there will be approximately 50 billion devices connected to the Internet by 2020 [4]. In this respect, we can say that there is a big demand for IoT applications since IoT has a wide range of applications such as intelligent transportation systems, smart home and building automation, medical and health systems, industrial management, energy systems, environment and infrastructure monitoring [5,6,7,8]. For understanding of IoT applications, IoT systems need to be divided into small components in view of tasks [2]. Thus, the first task of IoT systems is to identify and monitor the objects communicating with. The second task is to collect and process the information. In this structure, we have two main problems: identification and (secure) communication. Therefore, we focus on efficient and secure identification in IoT systems in this paper.

Identification is crucial to communicate among devices securely and properly. Radio-frequency identification technology (RFID) serves to identify objects automatically with electromagnetic frequency waves in IoT [9]. An RFID system consists of two components: the RFID tag which collects the information and transmits the collected data to the application and the RFID reader which stores the information [10]. Identification schemes used in RFID applications have some properties such as scalability, low memory requirement, communication and computational cost. In addition, an identification scheme has zero-knowledge property that does not allow an intruder verifier to hold communication [11]. CryptoGPS protocols which are of standard designed commonly use RFID applications [12,13]. CryptoGPS is a public key identification scheme which uses modular explementation in a multiplication group or a scalar multiplication in an additive group. Recall that after the quantum computer idea had been introduced, a quantum computer with two qubits was build. Then, the number of qubits increased. Nowadays, we know that Google and D-wave have 72-qubit and 2048-qubit quantum computers, respectively [14]. In addition, the systems whose security depends on integer factorization or discrete logarithm problems which are used in the identification schemes are called insecure due to Shor’s polynomial time algorithm in quantum computers [15]. For this reason, there is a big demand to construct quantum secure identification schemes. There are many classes since there is no known polynomial time algorithm to solve multivariate polynomial in any computational area. The cryptosystems based on multivariate polynomials are used in quantum secure areas [16,17,18].

In the literature, identification schemes based on multivariate quadratic polynomials as well as cubic ones have received interest since they are efficient for different platforms [19,20,21]. In [19], 3 and 5-pass zero-knowledge identification schemes based on multivariate quadratic (MQ) polynomials over a finite field were proposed. They defined bilinear functions as polar form of the MQ polynomial systems. In addition, they indicated whether or not it is able to build an efficient protocol using multivariate polynomials of degrees greater than two. In [20], 3-pass zero-knowledge identification scheme-based multivariate quadratic polynomials over a finite field by using the same bilinear functions were presented. However, they used a different way to divide secret key. Then, in [21], these identification schemes were improved in view of communication complexity by using new dividing techniques with the help of bilinearity of a polar form of the MQ function. Moreover, 3 and 5-pass identification schemes based on multivariate cubic (MC) polynomials over finite field were proposed. A new associated linear-in-one argument form, i.e., polar form of MC functions was defined and can be applied to a cubic version of the problem without changing bilinear form. The main difference between [19] and [21] is the degree of multivariate polynomial systems.

A different polar form is used with the same bilinear property. In [22], identification schemes based on multivariate polynomials over a finite field in the literature were surveyed. 3 and 5-pass identification schemes based on multivariate quadratic and cubic polynomials were given with the dividing technique of the secret key and polar form construction. In addition, all identification schemes based on multivariate polynomials were compared in view of memory requirements, communication length, and computation time. In [23], a new identification scheme based on multivariate quadratic polynomials was presented by using a different dividing technique of the secret key. Then, the proposed identification scheme was transformed to the signature scheme.

### 1.1. Motivation

In [21], efficient constructions based on multivariate polynomials of degree d≥4 are given as an open problem (see Section 2.1). The hardness of this problem comes from the nonlinear terms in f(x+y)−f(x)−f(y). In [24], a solution with a different perspective was proposed for the open problem. They denoted a polarization identity for a system of multivariate polynomials of any degree *d*. The function $G:{\left(F_{q}^{n}\right)}^{d}\to F_{q}^{m}$ is given as follows:

(1) $$G\left(r_{0},r_{1},\ldots ,r_{d-1}\right)=\sum_{i=1}^{d}{\left(-1\right)}^{d-i}\sum_{\begin{array}{c} S\subset \{0,\ldots ,d-1\}\\ |S|=i \end{array}}F\left(\sum_{j\in S}r_{j}\right).$$

By Equation (1), polar form of the system of multivariate polynomials in d−linear form was obtained. They also generalized identification protocol for multivariate d−degree polynomials with this perspective.

Variants of CryptoGPS are used in RFID applications. However, these protocols are not quantum secure. There is a need to construct identification schemes that are secure against quantum attacks.

### 1.2. Our Contribution

We propose a solution for the open problem with a different perspective. We generalize polar form for any degree of multivariate polynomials using bilinear functions. We define how the polar function will be when any degree of multivariate polynomials is used with Theorem 1. The idea comes from the bilinear functions used in both multivariate quadratic [19,20] and cubic [21] polynomials over a finite field. We present a generic identification scheme based on any degree of multivariate polynomials. In addition, we provide a comparison for the cryptoGPS standard and identification schemes based on multivariate polynomials. The generic identification scheme framework can be used to construct identification scheme for RFID applications due to the zero-knowledge property.

### 1.3. Organization

The rest of this paper is organized as follows. In Section 2, we give some definitions and then explain our solution proposal for the open problem. Moreover, we express our solution with examples and compare it to the other approach [24] for the open problem. We give a generic scheme for identification construction when using any degree of multivariate polynomials. Then, we compare curve-based cryptoGPS and identification schemes based on multivariate polynomial. The conclusion and future works are stated in Section 3.

## 2. A Novel Method for Polar Form of Multivariate Polynomials of Any Degree

In this section, we first review some definitions and notations. We recall the open problem given in [24]. Then, we express our method for polar form of multivariate polynomials and give examples. We compare our solution and the other approach [24].

### 2.1. Mathematical Background

Multivariate cryptography depends on solving systems of multivariate polynomials of degree *d* over a finite field. For d=2 or d=3, then it is called MQ or MC, respectively. The system of multivariate polynomials of degree *d* with *n* variables and *m* equations can be given as follows:

(2) $$\begin{array}{cc} f^{\left(1\right)}\left(x_{1},\ldots ,x_{n}\right)=\sum_{i}^{n}\cdots \sum_{j}^{n}f_{i\cdots j}^{\left(1\right)}\overset{d}{\overset{︷}{x_{i}\cdot \ldots \cdot x_{j}}} & +\ldots +\sum_{i=1}^{n}f_{i}^{\left(1\right)}\cdot x_{i}+f_{0}^{\left(1\right)}\\ f^{\left(2\right)}\left(x_{1},\ldots ,x_{n}\right)=\sum_{i}^{n}\cdots \sum_{j}^{n}f_{i\cdots j}^{\left(2\right)}\overset{d}{\overset{︷}{x_{i}\cdot \ldots \cdot x_{j}}} & +\ldots +\sum_{i=1}^{n}f_{i}^{\left(2\right)}\cdot x_{i}+f_{0}^{\left(2\right)}\\ \vdots & \\ f^{\left(m\right)}\left(x_{1},\ldots ,x_{n}\right)=\sum_{i}^{n}\cdots \sum_{j}^{n}f_{i\cdots j}^{\left(m\right)}\overset{d}{\overset{︷}{x_{i}\cdot \ldots \cdot x_{j}}} & +\ldots +\sum_{i=1}^{n}f_{i}^{\left(m\right)}\cdot x_{i}+f_{0}^{\left(m\right)}. \end{array}$$

where the coefficients ($f_{i\ldots j}^{\left(k\right)}$ and $f_{i}^{\left(k\right)}$ for 1≤k≤m) are in a finite field. Note that the MQ problem, which is NP-complete, can be defined as follows:

**Definition** **1.** *[15] Given m multivariate quadratic polynomials $f^{\left(1\right)}\left(x\right),\ldots ,f^{\left(m\right)}\left(x\right)$ as shown in Equation* (2)*, find a vector $\bar{x}=\left({\bar{x}}_{1},\ldots ,{\bar{x}}_{n}\right)$ such that $f^{\left(1\right)}\left(\bar{x}\right)=\ldots =f^{\left(m\right)}\left(\bar{x}\right)=0$.*

The hardness of MQ problem depends on the hardness of solving nonlinear equations over finite field [15]. In Definition 2, the polar form of the MQ function is given.

**Definition** **2.** *[19] Let $x\in F_{q}^{n}$, $f_{\ell }\left(x\right)$ be multivariate quadratic function for 1≤ℓ≤m and $F\left(x\right)=\left(f_{1}\left(x\right),f_{2}\left(x\right),\ldots ,f_{m}\left(x\right)\right)\in MQ\left(n,m,F_{q}\right).$ Then polar form of the MQ function is G(x,y)=F(x+y)−F(x)−F(y).*

**Definition** **3.** *Let G:A×A→B be a bilinear function. Then, G satisfies the following properties:*

- *G(x+y,z)=G(x,z)+G(y,z) and G(ax+y,z)=aG(x,z)+G(y,z)*
- *G(x,y+z)=G(x,y)+G(x,z) and G(x,ay+z)=G(x,z)+aG(y,z)*

*where x,y∈A, z∈B and a is a constant.*

**Remark** **1.** *The function G in Definition 2 is bilinear since $G\left(x,y\right)=\sum_{ij}a_{\ell ,i,j}\left(x_{i}y_{j}+y_{i}x_{j}\right)$, where x,y are n−dimension vectors. Because of the bilinearity of G:A×A→B, G(x+y,z)=G(x,z)+G(y,z), where x,y∈A and z∈B. Note that this is only valid for MQ systems. It is difficult to control the terms in d−degree of elements because the number of terms increases when higher-order functions are used.*

In Definition 4, polar form of MC function which is a natural extension of MQ ones is given.

**Definition** **4.** *[21] Let $x\in F_{q}^{n},$$F\left(x\right)=\left(f_{1}\left(x\right),f_{2}\left(x\right),\ldots ,f_{m}\left(x\right)\right)\in MC\left(n,m,F_{q}\right)$ and $f_{\ell }\left(x\right)=\sum_{i,j,k}a_{\ell ,i,j,k}x_{i}x_{j}x_{k}+\sum_{i,j}b_{\ell ,i,j}x_{i}x_{j}+\sum_{i}c_{\ell ,i}x_{i}.$$G=(g_{1},\cdots ,g_{m})$ is the polar form of F for ℓ=1,⋯,m, $g_{\ell }\left(x,y\right)=\sum_{i,j,k}a_{\ell ,i,j,k}\left(x_{i}x_{j}y_{k}+x_{i}y_{j}x_{k}+y_{i}x_{j}x_{k}\right)+\sum_{i,j}b_{\ell ,i,j}x_{i}y_{j}$ and $g_{\ell }\left(y,x\right)=\sum_{i,j,k}a_{\ell ,i,j,k}\left(y_{i}y_{j}x_{k}+y_{i}x_{j}y_{k}+x_{i}y_{j}y_{k}\right)+\sum_{i,j}b_{\ell ,i,j}y_{i}x_{j}$ where x,y are n−dimension vectors over $F_{q}^{n}$. Moreover, G is a bilinear function and is obtained from F(x+y)=F(x)+G(x,y)+G(y,x)+F(y).*

Now, we recall the open problem given in [21].

**Open** **Problem.** *In [19,20,21], identification schemes based on multivariate quadratic and cubic polynomials were given. In [21], an open problem was defined: Is there any general method to construct polar form of multivariate polynomials of degree d≥4 in an efficient way?*

By considering [19,20,21], we have polar forms of multivariate quadratic and cubic polynomials. By using bilinear functions, polar forms of multivariate polynomials of degree of d≥4 can be obtained.

### 2.2. Another Look At Open Problem

Our aim is to generalize polar form for multivariate *d*-degree polynomials over a finite field. In Theorem 1, we introduce the generalization of linear-in-one-argument for the function of degree d≥4 which is a modified construction derived from the MQ and MC. Note that by recursive approach, one can obtain the linear form.

**Theorem** **1.** *Let $F_{q}$ be a finite field, $x,y\in F_{q}^{n}$ and $F\left(x\right)=(f_{1}\left(x\right),f_{2}\left(x\right),\ldots ,f_{m}\left(x\right))$, $f_{\ell }\left(x\right)$ be any degree of multivariate polynomial. Let $P(x^{j},y^{i-j})$ be a permutation with $\left(\begin{array}{c} i\\ j \end{array}\right)$ elements which contains j times x and i−j times y in any positions for a vector with i elements. Then, the generalization of linear-in-one-argument form for the function of degree d≥4 is defined as F(x+y)=F(x)+G(x,y)+G(y,x)+F(y), where*

$$\begin{array}{c} G\left(x,y\right)=xy+\sum_{i=3}^{d}\left\{\begin{array}{cc} \sum_{j=1}^{\frac{\left(i-1\right)}{2}}P\left(x^{i-j},y^{j}\right) & if\;i\;is\;odd,\\ \sum_{j=1}^{\left(\frac{i}{2}-1\right)}P\left(x^{i-j},y^{j}\right)+\frac{P(x^{i/2},y^{i/2})}{2} & if\;i\;is\;even. \end{array}\right. \end{array}$$

*and*

$$\begin{array}{c} G\left(y,x\right)=yx+\sum_{i=3}^{d}\left\{\begin{array}{cc} \sum_{j=1}^{\frac{\left(i-1\right)}{2}}P\left(x^{j},y^{i-j}\right) & if\;i\;is\;odd,\\ \sum_{j=1}^{\left(\frac{i}{2}-1\right)}P\left(x^{j},y^{i-j}\right)+\frac{P(x^{i/2},y^{i/2})}{2} & if\;i\;is\;even. \end{array}\right. \end{array}$$

**Proof.**  Both Definition 2 and Definition 4 have the same structure in terms of the usage of the functions. In other words, G(x,y) is complement of G(y,x) in view of commutativity.

Our observation is that the polar form defined for the quadratic and cubic versions can be generalized for any degree of multivariate polynomial systems since G(x,y) and G(y,x) can be obtained by using this idea. We combine this observation with the complementary property of the function. The formula can be classified for any *z* by considering even or odd case. In this step, we have two cases:

1. When *i* is odd, it is obvious to compute G(x,y) and G(y,x) since the number of the outputs of the *P* permutation is odd and the number of *x* and *y* in the output of the *P* permutation is not equal.
2. When *i* is even, we need to consider whether the number of *x* vectors equals the number of *y* vectors. In this case, we need to take half of the existing terms to be complementary. For this reason, the even case is different from the odd case.

Then, the number of distinct elements in G(x,y) or G(y,x) is $2^{d}-d-1$ due to the Pascal triangle. To compute each part of G(x,y) or G(y,x), $2^{d}-d-1$ distinct elements having two vectors (only *x* and *y*) up to *d* vectors (i−j times *x* and *j* times y) are computed. For d=2 case, the permutation *P* permutes the positions of *x* and *y* in the element. Note that some terms are complement the other, i.e., G(x,y) is complement of G(y,x). This completes the proof. □

Now, we look at the following examples to compare our solution with the other approach [24]. In Example 1, we apply Theorem 1 to multivariate cubic polynomials (d=3 case) and show that this is the same in Definition 4.

**Example** **1** (bilinear polar form of degree 3)**.** *For d=3, we have*

F(x+y)=F(x)+G(x,y)+G(y,x)+F(y)

*By using Theorem 1, $g_{\ell }\left(x,y\right)=\sum_{i,j,k}a_{\ell ,i,j,k}\left(x_{i}x_{j}y_{k}+x_{i}y_{j}x_{k}+y_{i}x_{j}x_{k}\right)+\sum_{i,j}b_{\ell ,i,j}x_{i}y_{j}$ and $g_{\ell }\left(y,x\right)=\sum_{i,j,k}a_{\ell ,i,j,k}\left(y_{i}y_{j}x_{k}+y_{i}x_{j}y_{k}+x_{i}y_{j}y_{k}\right)+\sum_{i,j}b_{\ell ,i,j}y_{i}x_{j}.$ These $g_{\ell }\left(x,y\right)$ and $g_{\ell }\left(y,x\right)$ polynomials are same in [21] and in Definition 4.*

In Example 2, we compare bilinear polar form by using Theorem 1 with trilinear polar form given in [24]. Since the idea is totally different, we have distinct polar forms.

**Example** **2** (trilinear polar form)**.** *When we use trilinear functions for d=3, we have*

$$\begin{array}{ccc} F(x+y+z) & = & G(x,y,z)+F(x+y)+F(x+z)+F(y+z)-F(x)-F(y)-F(z) \end{array}$$

*According to [24], $g_{\ell }\left(x,y,z\right)=\sum_{i,j,k}a_{\ell ,i,j,k}\left(x_{i}y_{j}z_{k}+x_{i}y_{k}z_{j}+x_{j}y_{i}z_{k}+x_{j}y_{k}z_{i}\right)$ where x,y and z are n−dimension vectors over a finite field and ℓ∈{1,…,m}.*

From Examples 1 and 2, we see that fewer terms are needed to in the use of bilinear functions than trilinear ones. To compute F(s), by using bilinear function four functions are needed to be computed. However, by using trilinear function one needs seven functions. Note that the bilinear polar form is simpler and more controllable.

In Example 3, we apply Theorem 1 to multivariate quartic polynomials (d=4 case).

**Example** **3.** *Let d=4. Then, multivariate quartic polynomials system F is in the following form: $F\left(x+y\right)=\sum_{i,j,k,t}a_{\ell ,i,j,k,t}\left(x_{i}x_{j}x_{k}x_{t}+y_{i}y_{j}y_{k}y_{t}+x_{i}x_{j}x_{k}y_{t}+x_{i}x_{j}y_{k}x_{t}+x_{i}y_{j}x_{k}x_{t}+y_{i}x_{j}x_{k}x_{t}+x_{i}x_{j}y_{k}y_{t}+x_{i}y_{j}x_{k}y_{t}+x_{i}y_{j}y_{k}x_{t}+y_{i}y_{j}y_{k}x_{t}+y_{i}y_{j}x_{k}y_{t}+y_{i}x_{j}y_{k}y_{t}+x_{i}y_{j}y_{k}y_{t}+y_{i}y_{j}x_{k}x_{t}+y_{i}x_{j}y_{k}x_{t}+y_{i}x_{j}x_{k}y_{t}\right)+\sum_{i,j,k,t}b_{\ell ,i,j,k}\left(x_{i}x_{j}x_{k}+y_{i}y_{j}y_{k}+x_{i}y_{j}x_{k}+y_{i}x_{j}x_{k}+x_{i}x_{j}y_{k}+y_{i}x_{j}y_{k}+x_{i}y_{j}y_{k}+y_{i}y_{j}x_{k}\right)+\sum_{i,j}c_{\ell ,i,j}\left(x_{i}x_{j}+y_{i}y_{j}+x_{i}y_{j}+x_{j}y_{i}\right)+\sum_{i}d_{\ell ,i}\left(x_{i}+y_{i}\right)$.*

*By using Theorem 1, the linear-in-one-argument form of F is:*

(3) $$\begin{array}{c} G\left(x,y\right)=xy+P\left(x^{2},y\right)+P\left(x^{3},y\right)+\frac{P(x^{2},y^{2})}{2}. \end{array}$$

(4) $$\begin{array}{c} G\left(y,x\right)=yx+P\left(x,y^{2}\right)+P\left(x,y^{3}\right)+\frac{P(x^{2},y^{2})}{2}. \end{array}$$

*$P(x^{2},y)$ computed in Equation* (3)* is the complement of $P(x,y^{2})$ in view of variables given in Equation* (4)*, i.e., $P\left(x^{2},y\right)=\bar{P(x,y^{2})}$. In a similar manner, all permutation used in G(x,y) are selected as a complement of those used in G(y,x).*

### 2.3. Comparison

Now, we compare the proposed solution with d−linear form in terms of the required number of the functions in Table 1.

The number of partitions of *s* secret key is constant in the proposed method because of the bilinearity. However, in [24] it depends on the degree *d*. The number of *F* and *G* functions to compute F(s) in bilinear polar form is 2(r+1), where *r* is recursion number, whereas it is $2^{d}-1$ $\left(\left(\begin{array}{c} d\\ d \end{array}\right)+\left(\begin{array}{c} d\\ d-1 \end{array}\right)+\cdots +\left(\begin{array}{c} d\\ 1 \end{array}\right)\right)$ for d−linear form [24]. When F(s) is computed, one needs to calculate two *F* functions. In addition, one must compute two *G* polar form according to the proposed generalization form given in Theorem 1. Note that *r* is the loop number required to obtain each *G* polar form. For example, the secret key is divided into two parts as *x* and *y*. Then, we compute F(x+y) by using polar form given in Definition 4. According to the proposed solution, the recursive construction is used for the calculation of G(x,y). Let d=4 be the degree of multivariate polynomials. The recursion number is r=2 since we run G(x,y) function for d=4 and d=3. Then, G(x,y) function is obtained by adding quadratic terms. G(y,x) is computed similarly. Actually, we use a divide-and-conquer approach for computing G(x,y) and G(y,x) function. The number of *F* and *G* functions is 2(r+1) by adding the number of *F* functions. The number of *F* and *G* functions is the main factor to determine the computation time in the identification scheme based multivariate polynomial systems (for more details, see [20]). Note that for efficiency reasons, the number of *F* and *G* functions should be as small as possible due to the arithmetic operations. Memory requirements in bilinear form and d−linear form are 2m(r+1) or $(2^{d}-1)m$, respectively, where *m* is bit size of the output of *F* and *G* functions.

### 2.4. A Generic Identification Scheme Based on Multivariate Polynomials

**Figure 1:**
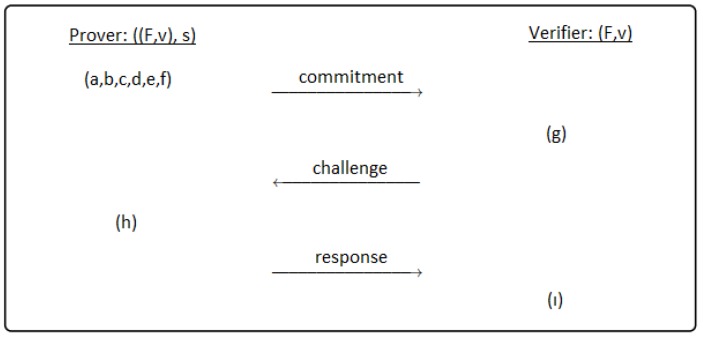
A 3-pass identification scheme.

In this section, we present a generic scheme for identification construction based on any degree of multivariate polynomials. The identification schemes are compared with the cryptoGPS standard. A 3-pass identification scheme is given in Figure 1.

A generic 3-pass identification scheme between prover and verifier has the following passes:

1. Commitment: The prover performs this pass.
  (a) The multivariate polynomial system *F* is constructed with *n* variables and *m* equations.
  (b) Choose randomly secret key $s\in F_{q}$ and generate public key v=F(s).
  (c) Divide secret key *s* into parts and sub-parts. Generate randomly the parts of *s*, not all of them. For example; let *s* be divided into *k* parts. According to dividing technique, the sub-parts of the secret key like $\left(r_{1},r_{2},\ldots ,r_{k-1}\right)\in F_{q}$ are generated randomly. Note that the secret key parts must be evaluated in *F* polynomial system. v=F(s) is satisfied with the help of polar form.
  (d) Compute the parts of the secret key, not chosen randomly at step *c*. Thus, all parts of the secret key are obtained before the commitment phase.
  (e) According to dividing technique and polar form, the commitment values $c_{1},c_{2},\ldots ,c_{i}$ are computed, where i∈N. The number of the commitment values is changeable according to the constructed scheme.
  (f) In this step, the prover sends the commitment values. Thus, the prover commits the verifier. At the end of this step, the first pass of the identification scheme is completed.
2. Challenge: At the second phase, all operations are performed by the verifier.
  (g) The verifier makes a challenge to the prover. For example; the verifier chooses a challenge value Ch∈{0,1,…,n−1} and sends to the prover.
3. Response: This phase is performed by the prover.
  (h) The prover sends the responses which belong to each challenge in order that the verifier computes the commitment values.
    At the end of the this step, the verifier carries out the following step and terminates the identification scheme as accepted or rejected.
  (i) The verifier computes the commitment values after the verifier receives the responses. Then the verifier compares these commitment values with the values which sent as commitment by the prover. If they are equal, the verifier accepts the prover’s commitment. Otherwise, the verifier rejects. When the verifier computes the commitment values, the verifier can compute the same commitment value with different parts of the secret key unlike the prover. Note that the same values are obtained by using polar form.

**Remark** **2.** *A zero-knowledge identification scheme provides us to restrict information which is sent from the prover to the verifier. When the verifier has any limited and any useless information, he/she can prove the prover’s commitments. An identification scheme has to satisfy soundness and completeness properties for security and zero-knowledge. If an identification scheme is completed, i.e., the verifier accepts the prover’s commitment, the identification scheme has completeness property [25]. If the verifier can not be deceived by the intruding prover (except negligible probability ϵ > 0), the identification scheme has soundness property [25]. One of the important criteria for identification scheme is impersonation probability. It is defined as the probability that an adversary would impersonate the prover without knowing the secret key.*

The proposed generic scheme depends on the *d*th degree multivariate polynomials. The key pair and communication length are calculated easily with only basic polynomial operations. However, curve-based cryptoGPS identification schemes, which commonly use IoT applications, require scalar-point multiplications. Note that we prove a generic identification scheme framework in Section 2.4. Thus, we do not have numeric parameters for any security level. However, the comparison is provided by identification schemes based on multivariate quadratic or cubic polynomials presented in [19,21,23] with curve-based cryptoGPS [26]. In Table 2, for 80-bit security level, the comparison is given by considering security attacks against quantum attacks, impersonation probability and parameter set including secret key, challenge and response sizes.

In Table 2, challenge indicates the bit size of challenge which is determined so that the verifier can obtain all commitment values for identification schemes based on multivariate polynomials. When we compare the identification schemes for the size of response, identification schemes given in [19,21] have small values. The main reason of small key sizes is the bilinear polar form of multivariate polynomials of degree d>1. If the identification scheme based on multivariate polynomials is well-organized, it has lower cost than cryptoGPS. Thus, it can be used IoT applications efficiently.

## 3. Conclusions

In this paper, we propose the generalization of linear in one argument form for the function of degree d≥4 and provide a solution for the open problem given in [21]. When the system of multivariate d−degree polynomials is used, we show how to construct the polar form by using a bilinear function. We present a generic framework to construct the identification scheme based on multivariate (cubic) polynomials. Then, we compare curve-based cryptoGPS and the identification schemes based on multivariate polynomials. We explain that the identification scheme based on multivariate polynomials can be used in quantum secure RFID applications. As a future work, new identification schemes based on multivariate *d*th polynomial systems can be constructed. Then, one can construct a signature scheme based on these identification schemes.

## Figures and Tables

**Table 1:** Comparison of the proposed solution and d−linear functions.

	Partitions	The Number of Functions	Memory Requirements
This paper	2	2(r+1)	2m(r+1)
d-linear [24]	*d*	$2^{d}-1$	$(2^{d}-1)m$

**Table 2:** Comparison of the identification schemes and cryptoGPS.

	cryptoGPS [26]	MQ-IDS [19]	MQ-IDS [23]	MC-IDS [21]
Secret key	160 bits	84 bits	84 bits	84 bits
Challenge	848 bits	104 bits	60 bits	146 bits
Response	1088 bits	248 bits	896 bits	248 bits
Impersonation probability	$2^{-32}$	$2^{-30}$	$2^{-30}$	$2^{-30}$
Quantum secure	No	Yes	Yes	Yes
